# HOW CAN WE SOLVE THE LONELINESS PROBLEMS WITH INTERGENERATIONAL DIGITAL DIVIDE?

**DOI:** 10.1093/geroni/igad104.2726

**Published:** 2023-12-21

**Authors:** Yeseul Lee, Si Young Song, Susanna Joo, Hyoun Kyoung Kim

**Affiliations:** Yonsei University, Seoul, Republic of Korea; Kyung Hee University, Seoul, Republic of Korea; BK21. Symbiotic Society and Design, Seoul, Republic of Korea; Yonsei University, Seoul, Republic of Korea

## Abstract

As Korean society is rapidly digitalizing and aging, ’the intergenerational digital divide’ due to a large gap in digital skills between the younger and older generations is rising social issues. Lack of digital skills among older adults is likely to make them feel isolated with limited opportunities for social integration in a highly digitalized society. This study examined the extent to which digital skills and intergenerational integration were differently associated with loneliness in Korea between the younger and older generations with the aim of informing efforts to reduce loneliness. The data was collected through an online survey from December 2021 through January 2022. The sample consisted of 315 Korean older adults aged 65 or above and 322 young adults in their 20s. The loneliness with 11 items from Loneliness Scale, digital skills with 4 items from Digital Citizenship Scale, Intergenerational Integration Scale with 30 items and a binary generation variable (young adults=0, older adults=1) were measured. Results from a serial mediation analysis showed that, compared to younger adults, older adults had lower levels of digital skills, which led to low intergenerational integration, which in turn affected loneliness. Findings from two simple mediation paths showed that only digital skills had a significant single mediating role in the association between generation and loneliness but intergenerational integration did not. This study suggests that enhancing digital skills with providing opportunities for online intergenerational interaction can decrease loneliness and associated issues, especially for older adults.

